# 

**Published:** 1888-07

**Authors:** 


### OUR COLLABORATORS.

We have the pleasure of announcing to our readers that we have added to our list of collaborators the names of three intelligent and prominent medical gentlemen, to-wit: Dr. P. R. Cortelyou, of Marietta, Georgia, formerly of Brooklyn, New York, a prominent member of the Society of the County of Kings, a fine writer and an active progressive practitioner; Dr. K. P. Moore, of Macon, Georgia, an able practitioner and writer, and a well-known working member of the Medical Association of Georgia, having been chosen as Vice-President, Orator, Treasurer, President, and now the Secretrry of the body; Dr. Julian J. Chisolm, of Baltimore, a distinguished specialist of the eye, ear and throat in that city, a great surgeon, a superior writer, and a gentleman of world-wide reputation in the profession. These medical gentlemen have all promised to write one or more articles for our journal during the present year.

Our other collaborotors, Prof. J. McF. Gaston, Prof. W. D. Bizzell, Prof. W. P. Nicolson, Prof. G. G. Roy, Prof. A. G. Hobbs, Dr. W. S. Elkin, Dr. W. A. Crow, and Prof. J. Thad. Johnson, are well-known, able and prominent professors and lecturers in the Southern Medical College of this city. Dr. E. van Goidtsnoven is a learned and progressive practitioner, and a good writer, who is rapidly rising to prominence in the profession.

RECEIPTS will appear in our next.

